# Thyroid metastasis of clear renal cell carcinoma: a case report and review of the literature

**DOI:** 10.11604/pamj.2024.49.26.43171

**Published:** 2024-10-03

**Authors:** Flores Paños Alberto, Marin Martinez Luis, Espinosa Sanchez Alberto, Georgios Kyriakos, Antonio Javier Rios Vergara, Hernandez Alonso Enrique

**Affiliations:** 1 Endocrinology and Nutrition Department, Hospital General Universitario Santa Lucía, Murcia, Spain,; 2 Pathology Department, Hospital General Universitario Santa Lucía, Murcia, Spain

**Keywords:** Clinical endocrinology, clear renal cell carcinoma, thyroid nodule, case report

## Abstract

The thyroid is a rare site for finding tumor metastases. Renal, colorectal, pulmonary, and mammary origin are the most frequent primary neoplasms. Clinical suspicion, early diagnosis, and active surveillance are important during follow-up. Thyroid ultrasound and fine needle aspiration thyroid ultrasound are crucial during follow-up. We present a case of a 66-year-old male who was referred to our Endocrinology and Nutrition Department of the Hospital General Universitario Santa Lucía due to a multinodular goiter. The patient had no symptoms of hyperthyroidism or hypothyroidism. No weight loss or constitutional syndrome was reported. The patient was suffering from a renal clear cell carcinoma with T3aNxM0 stage operated on using a nephrectomy technique in 2012. In a new follow-up, a positron emission tomography-computed tomography (PET-CT) scan was conducted and a multinodular goiter was found with an increase in size and metabolism at the expense of a right thyroid nodule and thyroid ultrasound and fine needle aspiration thyroid ultrasound was requested with the diagnosis of renal cell carcinoma metastasis. We present a rare case report since both metastases (thyroid and pulmonary) could be surgically intervened with curative intent and a review of the literature. This case emphasizes the importance of considering a metastatic origin when finding a thyroid nodule in a patient with a previous history of clear renal cell carcinoma even years after treatment with curative intent.

## Introduction

In spite of its good vascularization, the thyroid is a rare site for the finding of tumor metastases, which are identified in around 1.4%-3% of thyroidectomies. These thyroidectomies resulted in malignant results. However, these tumor metastases may reach up to 1.9%-25% in clinical autopsies [1-5]. The primary neoplasms that most frequently metastasize have renal, colorectal, pulmonary, and mammary origin [1,4,6]. Among these neoplasms, renal origin is the most frequent with up to 48% in the literature reviewed [1,6].

Renal cell carcinoma (RCC) represents 90% of all renal tumors and about 3% of all malignant tumors in adulthood [1]. These tumors are characterized by their high vascularity and great clinical variability, and may behave unpredictably, causing distant metastases years after the primary tumor. The average survival rate of patients suffering from RCC thyroid metastases is 50% within 5 years after diagnosis [7]. This case report is rare first of all because of the form of presentation, more than 10 years after the primary renal tumor. It first manifested as pulmonary and paratracheal nodules that were operated on 5 years before the thyroid metastases. After the finding of thyroid nodules, a pulmonary metastatic origin was thought to be the cause but the anatomopathological was compatible with renal origin. This is a rare case since both metastases (thyroid and pulmonary) could be surgically intervened with curative intent.

## Patient and observation

**Figure 1:**
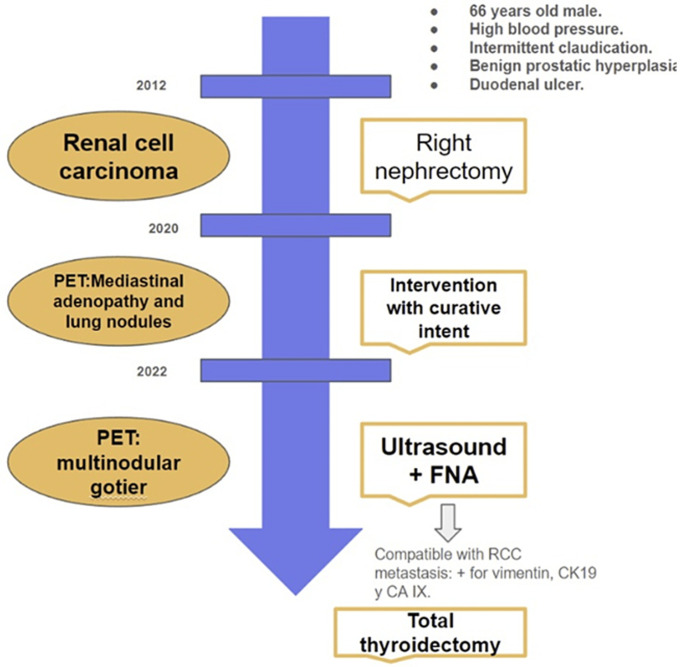
patient's chronological evolution

**Patient information:** Figure 1 is a summary of the chronological evolution of the patient. A 66-year-old male was referred to our Endocrinology and Nutrition Department of the Hospital General Universitario Santa Lucía due to a thyroid nodule. As the most remarkable medical report states, the patient was suffering from a renal clear cell carcinoma with T3aNxM0 stage. The patient was diagnosed and operated using nephrectomy technique in 2012. The patient had been undergoing check-ups since then. The last check-up in 2020 was a PET-CT scan, which resulted in a right paratracheal mediastinal adenopathy and pulmonary nodules in the right upper lobe. These are possibly related to malignant lesions with low affinity for 18f-FDG. In 2021 he underwent surgery to treat this 5 cm mediastinal tumor, which was in the retrocaval pretracheal space, and two hilar lesions in the right upper lobe. A new post-surgical PET-CT scan was conducted in which there was no clear evidence of macroscopic malignant disease related to 18f-FDG. After this result, the decision in order not to initiate adjuvant treatment was made. In 2022 a new follow-up PET-CT scan was conducted in which there was no evidence of macroscopic pathology in the pulmonary region. However, a multinodular goiter was found with an increase in size and metabolism at the expense of a right thyroid nodule.

**Clinical findings:** the patient only presented a slight discomfort when swallowing. The patient had no symptoms of hyperthyroidism or hypothyroidism. No weight loss or constitutional syndrome was reported. On physical examination, the patient had a palpable grade 1b goiter. There were no palpable cervical lymphadenopathies.

**Figure 2:**
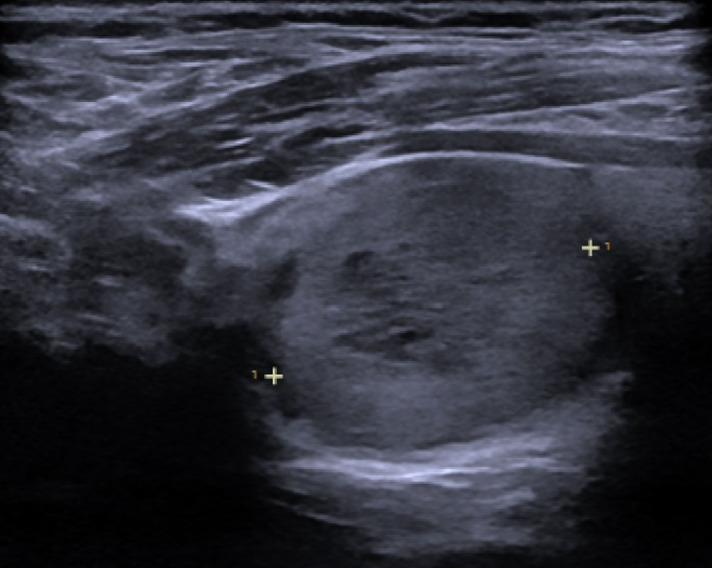
thyroid ultrasound: hypoechogenic, ovoid, solid thyroid nodule with well-defined margins

**Diagnostic assessment:** a blood test showed no signs of hyperthyroidism or hypothyroidism, with negative thyroid autoimmunity. With this clinical picture, the first suspicion was thyroid cancer, a benign thyroid nodule or a metastasis of renal cell carcinoma, so a thyroid ultrasound was requested. A fine needle aspiration thyroid ultrasound (FNA) was requested according to these results. The thyroid ultrasound showed two 2.3 x 2.3 x 2.7 cm and 1.8 x 1.8 x 1.8 cm (TIRADS-4) hypoechogenic with ovoid morphology, solid, and with well-defined margins nodules (Figure 2).

**Figure 3:**
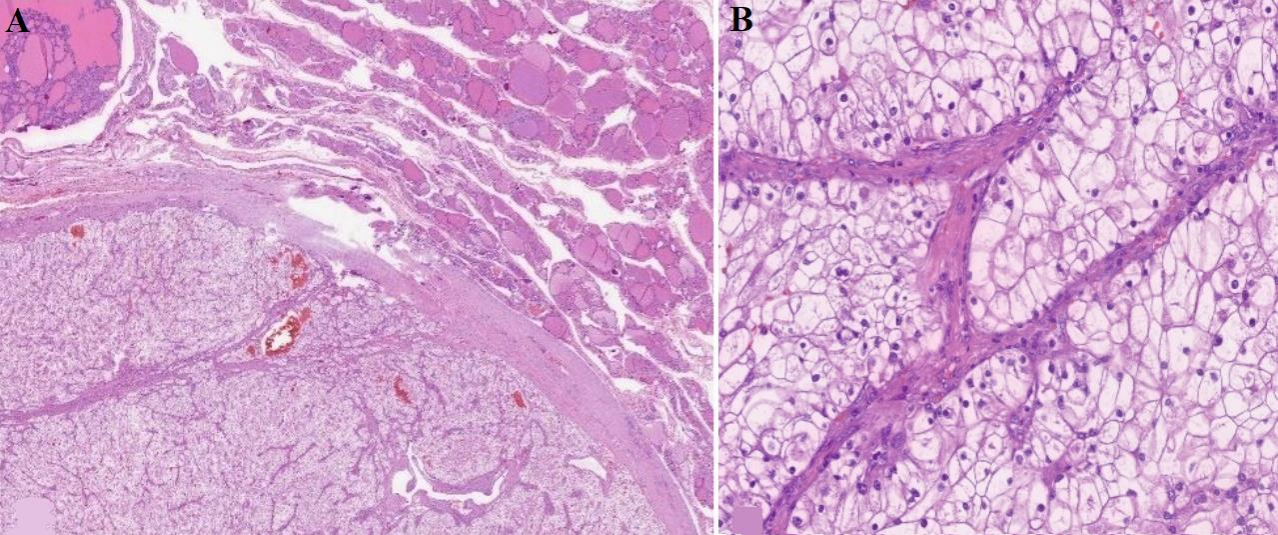
hematoxylin-eosin image of the thyroid lesion: A) a neoplastic lesion well delimited by fibrous connective tissue that is adjacent to thyroid parenchyma with medium-small follicles covered by a layer of cells without colloid atypia inside; B) the lesion is composed of a neoplastic population of cells with broad whitish cytoplasmic with rounded central nuclei, with small nucleoli, there is no evidence of irregularities or cellular atypia, the cells are surrounded by thin fibrovascular septa that partially compartmentalize the lesion

**Figure 4:**
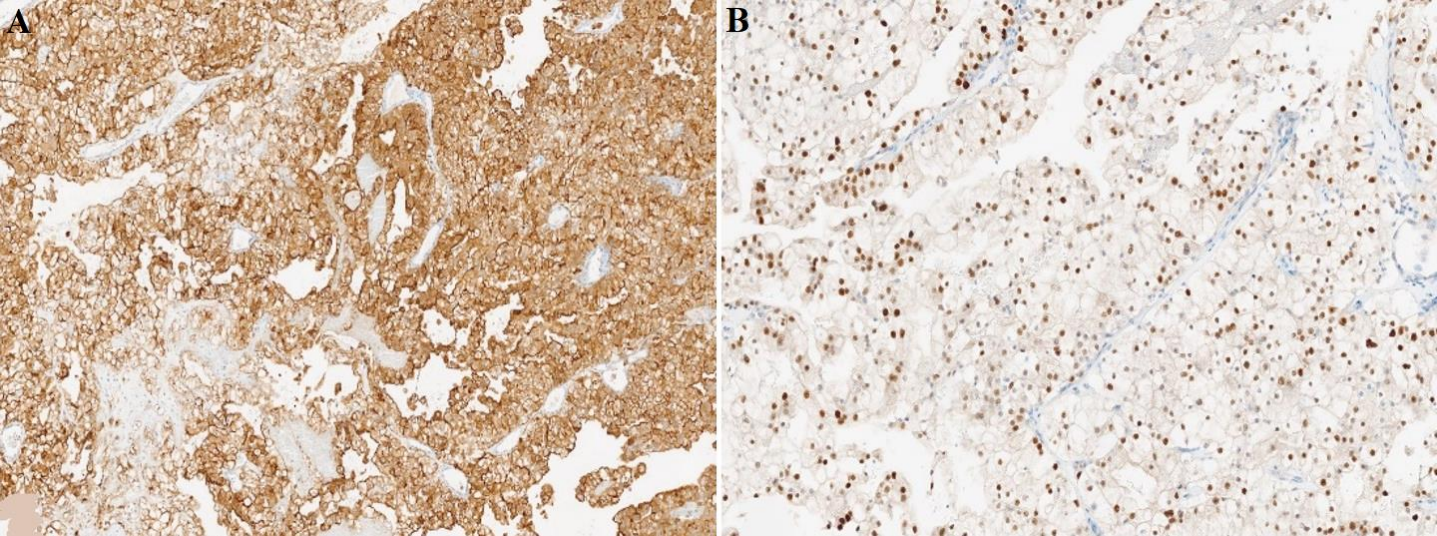
immunohistochemistry techniques: A) CD10 marker; the image shows intense positivity for the membrane marker CD10 in all tumor cellularity, compatible with the renal origin; B) PAX8 marker; the image shows intense nuclear positivity for the PAX8 marker, this staining is compatible with a renal origin, among other origins

It was decided to conduct an FNA test of the nodules, which showed anatomopathological results compatible with metastasis of renal clear cell carcinoma (Figure 3). Abundant isolated tumor cellularity in small groups is shown. This tumor is formed by cells with large clear cytoplasm and medium-sized nucleus that do not show clear changes compatible with papillary thyroid carcinoma, resulted positive in CA IX, vimentin, PAX8, CD10, and CK19 (Figure 4).

**Therapeutic intervention:** a total thyroidectomy was conducted with curative intent because it was a single thyroid metastasis and it was decided not to administer adjuvant treatment. The anatomopathologic analysis showed large cells with clear cytoplasm. They were separated by a thin fibrovascular septum showing high vascularization with hematic foci.

**Follow-up and outcomes:** the patient had no immediate or long-term postoperative complications. The patient is currently under active surveillance and at the time of writing this article has not presented new symptomatology or signs of recurrence. During follow-up, annual thyroid ultrasounds and PET-CT every two years are performed together with analytical control with TSH, T4, and thyroglobulin levels.

**Patient's perspective:** throughout the diagnostic process the patient was informed of all diagnostic possibilities and possible treatments available depending on the results. The patient felt confident about the diagnostic and therapeutic approach followed in his case. All his doubts about the surgical intervention and the necessary follow-up tests were resolved.

## Discussion

Renal cell carcinoma (RCC) is the most common subtype of renal neoplasm and accounts for approximately 3-4% of all malignant tumors in adulthood. It has a peak incidence between the sixth and eighth decades of the life of a patient [8]. Distant metastases with an incidence of 1-4% are frequently found, both synchronously and metachronously. These distant metastases may occur decades after the diagnosis of the primary tumor [8].

The thyroid gland is not a common place to find metastases with an incidence between 1.4%-3%. This percentage may increase up to 25% in clinical autopsies [1,4]. Despite its high vascularization, the pathophysiological mechanisms that reduce the probability of finding metastases in the thyroid are currently unknown [1]. Several theories have been exposed in this regard. In 1931 Willis [5] proposed that the rapid arterial flow in the thyroid together with the environment, rich in oxygen and iodine, made the adhesion and proliferation of malignant cells difficult. The alteration of these regulatory mechanisms in the thyroid gland would increase the probability of tumor cells nesting in the thyroid [1,5]. Despite this low incidence, the thyroid is the most frequent organ where RCC metastases are found, followed by the lung, bone, and liver [8-10].

These metastases are most frequently present metachronously, with an average latency of several years. According to the study by Chung *et al*. [1] was 5.8 years after diagnosis of the primary tumor, and 9.4 years in the study by Heffess *et al*. [7]. In our patient, there was an interval of 10 years until the thyroid metastasis was found. It is not uncommon to find distant metastases in several organs during the follow-up of a patient with RCC, occurring in up to 75% of patients in the study by Khaddour K *et al*. [10]. Our patient had distant pulmonary and mediastinal metastases prior to thyroid metastases during follow-up. It is impossible to clinically distinguish a thyroid metastasis from a primary tumor, so in the presence of a history of RCC, it is acceptable to treat the lesion as if it were metastatic [6]. These lesions may present asymptomatically or more characteristically as thyroid enlargement with palpable nodules, goiter, and compressive symptoms, such as dysphagia or dyspnea [6,9].

Regarding diagnosis, conventional imaging tests, such as thyroid ultrasound, CT, and magnetic resonance imaging (MRI), cannot distinguish this entity from neoplasms of thyroid origin. They are usually present as a solid, well-defined hypoechogenic nodule when ultrasound is conducted and as cold nodules in the PET-TC scan [4]. Fine needle puncture-aspiration is the diagnostic procedure for the pre-surgical approach. However, it is common to find a high false negative rate of up to 28.7% [4].

When conducting the immunohistochemical analysis, RCC markers have resulted in a positive CD10, vimentin, and cytokeratin. Negativity thyroid, such as TTF-1, calcitonin, and thyroglobulin, were also common. In our case, the markers CA IX, vimentin, and CK 19 were positive [4,6].

The best therapeutic approach with curative purposes when the metastasis is isolated is thyroidectomy, either partial or total, depending on the characteristics of each patient and the preferences of the surgical team. It shows an average survival rate with both treatments [3]. In the case of disseminated disease, surgical intervention is conducted when the patient presents compressive symptoms [3]. The 5-year survival average rate after surgery is estimated to be between 30% and 60%. However, the presence of metastases in the thyroid is an indirect sign of disseminated disease [10].

## Conclusion

Thyroid metastases are a rare entity, although not uncommon. The most frequent primary tumor is RCC. Metachronous presentation several years after the primary tumor is frequent so long time follow-up in patients with RCC is crucial. Early diagnosis and prompt surgical intervention through comprehensive diagnostic approaches are required. Diagnostic imaging tests cannot distinguish these lesions from tumor lesions originating in the thyroid, so the diagnosis is obtained by FNA and pathological analysis of the lesion. The therapeutic approach is based on thyroidectomy, total or partial in the case of a single metastasis. In conclusion, this case emphasizes the importance of considering a metastatic origin when finding a thyroid nodule in a patient with a previous history of RCC even years after treatment with curative intent.
